# Validating infant and maternal mortality reporting in Doddaballapur taluk of Bangalore Rural district – a pilot study

**DOI:** 10.1186/1753-6561-6-S5-O23

**Published:** 2012-09-28

**Authors:** Mohan Raju

**Affiliations:** 1 Karnataka Health Systems Resource Centre, Bangalore, India

## Introduction

Infant and maternal deaths are highly under-reported in the state. Incentives are given to the community to encourage reporting of infant and maternal deaths but this has not helped much. Consequently infant mortality rate (IMR) and maternal mortality ratio (MMR) reported by various districts are low and are not comparable to surveys such as the sample registration survey (SRS) or national family health surveys. This is affecting the quality of the planning undertaken at districts and state. The IMR reported by districts range from 8 to 20 deaths per 1000 live births, whereas according to SRS 2010 it is 38 per 1000 live births for Karnataka. Similarly MMR reported by districts is usually less than 100 maternal deatths for 100,000 live births, while by SRS 2007-09 it is 178 for Karnataka.

## Methods

We conducted a pilot study in Doddaballapur taluk of Bangalore Rural district. A house-to-house survey was got conducted in the taluk by Accredited Social Health Activists (ASHA) in the rural area and Anganwadi workers (AWW) in urban areas using a predesigned format. They were trained for conducting house visits and collection of data. The reference year taken was 2010-11 (April 2010 to March 2011). Every house was visited by the field investigators, who collected details of the deliveries, still-births, live births, infant deaths and maternal deaths for the reference period. This was supervised by the taluk health officer of Doddaballapur taluk. The data so collected were compared with the departmental data committed by taluk health officer for the year 2010-11. They were also triangulated with the vital statistics, if available from department of economics and statistics.

## Results

The results are summarised in Table 1 below.

**Table 1:** Data on deliveries, stillbirths, live births, infant and maternal deaths from three sources

*Events*	*Taluk routine data*	*Survey*	*Department of econ. & statistics*
Deliveries	4508	3112	Under compilation
Stillbirths	19	16	Under compilation
Live births	4489 (3 twins)	3099 (3 twins)	3011
Infant deaths	40(including deliveries of 2009-10) 32 exclusive for 2010-11	45 (for 2010-11)	Not available
Maternal deaths	6	6	Not available

Considering the accuracy of the civil registration system (CRS) to be 97% for births it is found that the number of live births as recorded by CRS and as obtained by survey are matching while the live births reported by the taluk are highly exaggerated. Similar is the case with deliveries. But number of stillbirths are comparable and maternal deaths are identical by survey and taluk data.

## Discussion

The possible reasons for over reporting of live births (deliveries) could be due to duplication at various levels. In the year 2010-11, Karnataka state followed area-based reporting for first four months and facility-based reporting for the remaining eight months. Hence the data is a mixture of area- and facility-based reporting.

Reasons for under-reporting of infant deaths were largely due to facility-based reporting by the institutions. Based on this study following recommendations were given to the state.

• Periodically check the data reported by the health department along with statistics of Department of Economics and Statistics;

• Encourage prompt reporting of infant and maternal deaths by community through better awareness generation;

• Audit every stillbirth (as infant deaths are reported camouflaged as stillbirths);

• Make reporting mandatory in private facilities and collect these data promptly (Underreporting in urban areas is mainly from private facilities).

## Competing interests

None declared

## Funding statement

None declared

